# Audience participation fighting game: Exploring social facilitation for an enhanced APG experience

**DOI:** 10.1016/j.heliyon.2023.e23967

**Published:** 2024-01-02

**Authors:** Pujana Paliyawan, Ruck Thawonmas, Kingkarn Sookhanaphibarn, Worawat Choensawat

**Affiliations:** a Ritsumeikan Center for Game Studies, Ritsumeikan University, Kyoto, Japan; b College of Information Science and Engineering, Ritsumeikan University, Japan; c Multimedia Intelligent Technology Lab, Bangkok University, Thailand

**Keywords:** Audience participation games, Game live streaming, Game AI, HCI

## Abstract

This paper discusses the popularity of live streaming video games and its potential to address psychological challenges, especially during the COVID-19 pandemic. An audience participation game (APG) that involve massive audiences in gameplay, blurring the lines between viewers and players, is introduced. The game highlights the dynamic adjustment of AI character strengths based on audience inputs, specifically cheering and jeering. The study examines factors that influence user experience (UX) and activeness in APG. System evaluation includes comprehensive AI testing, consisting of 500,000 one-minute game rounds, and a user experiment involving 82 participants. UX assessment is conducted using a pairwise preference, four-alternative forced choice (4-AFC), version of the Game User Experience Satisfaction Scale (GUESS). Finally, the paper concludes by offering guidelines and hypotheses for future research in the field of APGs.

## 1 Introduction

Live streaming video games, particularly on platforms like Twitch, have gained immense popularity [12]. Twitch has cultivated a global community through user-generated live streaming, offering live social interaction unlike other platforms [5]. The number of Twitch broadcasters and users has significantly increased, especially during the COVID-19 pandemic when social distancing became prevalent: the number of Twitch broadcasters and users has increased rapidly to 9 and 2.84 millions–about 4 times from 2017 to 2021.^1^

The World Health Organization (WHO) has highlighted the mental health crisis caused by COVID-19, emphasizing the importance of social contact through online channels, games, and social media.^2^ Therefore, in this era, live streaming games on platforms like Twitch have evolved beyond entertainment to become a potential solution for helping individuals cope with psychological problems. Twitch facilitates community building by providing social connectivity, interactivity, and opportunities for content discovery [23]. Live-streams serve as virtual places that foster multidimensional relationships between streamers and audiences, establishing communities with a sense of connection [28]. By leveraging these features, live streaming games offer more than just entertainment, but also a means of addressing psychological challenges in the current era.

In addition to live streaming games, Audience Participation Games (APGs) play a crucial role in fostering social connectivity by integrating large audiences into the gameplay [22]. APGs blur the boundaries between viewers and players by allowing audiences to actively impact the game [22], [24]. By engaging viewers, APGs have the potential to create authentic social connections, transforming one-way interactions into bidirectional social experiences similar to interacting with friends [29]. The level of audience participation in APGs is influenced not only by the immersive content but also by the immediacy of interactions and the social nature of the experience [9]. These interactions go beyond mere social interactions and become integral elements of the overall play experience [4]. In the context of the current social-distancing era, where individuals seek alternatives to in-person experiences, APGs offer a novel way for audiences to engage, although the potential design possibilities in this domain have yet to be fully explored [24].

While existing APGs can facilitate an enjoyable interaction between players and their audiences, there is a noticeable gap in the consideration of natural interaction. For instance, in Twitch Plays Pokémon [21], viewers can input message commands to vote on actions, influencing the AI player's in-game decisions, such as which attack skill a Pokémon should use. This approach is also prevalent in other games, like Legend of Dungeon: Masters [22] and JUSTIN [17]. In essence, these interactions primarily revolve around direct voting for actions, where the audience explicitly instructs the player or in-game bot on what to do.

In this paper, our focus is to explore more natural interactions within the context of APGs. We aim to shift away from direct commands and instead encourage the audience to engage in a manner akin to the experience of watching traditional streams. This approach creates a more immersive and organic gaming experience where the audience can cheer and jeer, significantly enhancing the overall interaction. Our research introduces a unique audience participation fighting game that leverages the concept of social facilitation [34], wherein an individual's performance is influenced by the presence of others. In this game, the strengths of AI characters are dynamically adjusted based on audience inputs. Our study investigates previously unexplored territories, delving into the factors that impact user experience (UX) and engagement in the realm of APGs. The findings underscore the influence of different gameplay modes (competitive and collaborative) and various types of audience interaction (cheering and jeering) in shaping the future of APGs.

This paper presents several contributions in the field of audience participation games (APGs), including:

• The development of the first audience participation fighting game that does not require modifications to the game itself, but instead adjusts the game's AI based on audience inputs.
• The first study examining differences in user experience (UX) and engagement in APGs, comparing competitive and collaborative game modes, as well as analyzing the impact of cheering and jeering interactions. The results of this study provide insights, directions, and hypotheses for future research in the field.
• The introduction of a 4-AFC (Four-Alternative Forced Choice) version of the Game User Experience Satisfaction Scale (GUESS) [20], a questionnaire-based assessment tool for evaluating UX. This version follows a pairwise preference protocol and draws from previous research in the field [31], [32], [3].

## 2 Literature review

This section begins by summarizing previous studies on social facilitation and their main findings and implications. It then introduces the concept of audience participation games (APGs) and discusses the limitations and challenges associated with these games. The paper also reviews previous research on audience experience and game design specifically in the context of APGs.

Additionally, it presents detailed information about FightingICE, a platform used in the study, and discusses previous research conducted on this platform. To provide a comprehensive overview, Table 1 is included, offering a summary of the comparisons made in the study.

**Table 1:** Comparison of the proposed work to existing works over seven dimensions: **[A]** Social Facilitation, **[B]** Many Audiences (>2), **[C]** APG (take audience inputs), **[D]** Study Audience Experience, **[E]** Cheer vs Jeer, **[F]** Competitive vs Collaborative, **[G]** AI.

	APG-related			Game-UX-related			AI
Comparison works	A	B	C	D	E	F	G
[1], [2], [13], [25], [26]	✓	X	X	X	X	X	X
[11]	✓	✓	X	✓	✓	X	X
[15]	✓	✓	X	X	X	X	X
[8]	✓	X	X	X	X	X	✓
[21], [22]	X	✓	✓	X	X	X	X
[17]	X	✓	✓	✓	X	X	X
[6], [7]	-	X	X	✓	X	X	X
[14]	-	✓	✓	✓	X	X	X
[16]	✓	X	✓	✓	X	X	X
[27]	-	X	X	X	X	✓	✓
[33]	X	X	X	X	X	X	✓
[18]	✓	X	✓	X	X	X	✓
**Proposed system**	✓	✓	✓	✓	✓	✓	✓

✓: yes, X: no, -: unclear or undefined.

### 2.1 Social facilitation

Social facilitation is a concept that has been studied for many years, with roots dating back to the late 18th century. One of the earliest studies by Norman Triplett in the late 1800s found that individuals performed better when competing with others, leading to the publication of a significant paper on social facilitation [25], [26]. This study sparked further research into understanding how the presence of others can influence individual performance, not only as competitors but also as observers and audiences. In 1920, Floyd Allport defined social facilitation as the phenomenon where individuals exhibit different performance levels when they are simply in the presence of others. Since then, researchers have continued to delve into the complex impact of social facilitation on human behavior and performance [2].

In the context of games, social facilitation has been explored in various studies. In 2014, Kappen et al. conducted the first study on the influence of co-located audiences on player experience [11]. While subsequent studies have been conducted in this area, there is currently no known study specifically focusing on Audience Participation Games (APGs). Most of these studies primarily examine the player's perspective. For instance, Kimble et al. examined player performance under audience scrutiny and found that players performed worse when observed during complex games [13]. Bowman et al. reported an increase in player performance when playing an easy version of a first-person shooter game in the presence of a real audience [1]. Lin et al. studied social interactions between audiences and players in a game arcade center environment [15]. Emmerich et al. investigated the impact of real observers and virtual agents on player experience and performance across four different games [8]. It is worth noting that many of these studies used controlled sets of audiences, including experimenters, rather than real audiences.

Social facilitation is a key concept underlying the proposed Audience Participation Game (APG). The game is a fighting game where two players, either human or AI-controlled, engage in combat. The unique aspect of the game is the introduction of an AI character whose strength is dynamically adjusted based on audience inputs. The system is evaluated with a large audience group in a real streaming environment. Furthermore, there are plans for future research to explore social facilitation by conducting a study where a human player competes against an AI opponent.

### 2.2 Audience Participation Games (APGs)

One notable early study on Audience Participation Games (APGs) was conducted by Seering et al. [22]. They presented two prototype games (FPS and Racing) with four different variants, exploring various methods of interaction. Twitch Plays Pokémon [21] emerged as a prominent example of an APG, inspiring the development of other games in this genre. For instance, Legend of Dungeon: Masters^3^ allows audiences to support the player by sending powerful weapons or unleashing challenging adversaries [22]. Another APG called JUSTIN was created to collect image description data through game live streaming [17].

A common limitation observed in many APGs is their reliance on chat channels for data transmission. This approach often imposes restrictions on the number of messages and can lead to a cluttered chat room. To overcome these limitations, we propose a new architecture that utilizes a web server with an SQL database. Additionally, previous research on APGs has not extensively explored the effects of different types of interactions within these games, indicating a gap in the existing knowledge.

### 2.3 Audience experience

Several recent studies have shed light on the audience's experience in social gaming situations, highlighting their importance within the overall gaming context. Downs et al. [6], [7] emphasized the need to better understand the experiences of audience members, particularly when their involvement extends beyond mere observation. They found that informing audiences about turn-taking in gaming sessions alters their experience. Lessel et al. [14] explored the enhancement of communication channels in video game live-streams, enabling audiences to feel more influential and engaged, but their design was specific to turn-based games. Maurer et al. [16] conducted a study using a game with gaze detection, comparing different conditions where the observer's gaze influenced the game experience for both players and audience members. They observed that integrating onlooker gaze changed the gaming experience. Similarly, our work focuses on user experience (UX) but involves comparing different designs for audience participation.

In terms of designs for audience participation, our study considers the following aspects:

**Cheer vs Jeer:** Kappen et al. [11] examined the impact of silent, positive, and negative audiences on the game experience of players. However, their study did not involve audience input to the game, the audiences were co-located, scripted comments were used for positive and negative audiences, and the focus was on the player. In contrast, our work focuses on the user experience (UX) of audiences in audience participation games (APGs) within a real streaming environment.

**Competitive vs Collaborative:** Wehbe et al. [27] explored the comparability of different social conditions (e.g., social cooperative, competitive, multiplayer environments, or computer-controlled single-player environments) and their impact on player experience. However, their study was conducted in a co-located setting, involving only two players and no audience in the tested game.

### 2.4 FightingICE and AIs

FightingICE^4^ is an open-source tool used for developing fighting game AI and hosting AI competitions. Many successful AI implementations (e.g., [33]) in these competitions have utilized Monte Carlo Tree Search (MCTS) techniques, with numerous MCTS-based AIs shared within the FightingICE community. For instance, Yoshida et al. [33] developed the MctsAi, which demonstrates the application of MCTS in fighting game AI development and is available on the FightingICE website.^5^

In our recent project [18], we developed an audience participation game (APG) version of FightingICE, integrating a generalized Believable Entertaining AI (gBEAI) powered by Monte Carlo Tree Search (MCTS). The gBEAI dynamically adjusts its strength to create engaging gameplay while maintaining human-like behavior, guided by the social facilitation parameter *F*, ranging from -1 to 1. With a value of -1, the AI aims to win against the opponent by a hit-point (HP) difference of 120, while a value of 1 prompts the AI to deliberately lose by the same HP difference. Unlike previous work that manually set *F*, we introduced dynamic adjustments based on audience interactions. Audiences can actively participate by clicking a cheer button to enhance their team's AI or a jeer button to weaken the opponent AI, adding an interactive element to the APG experience.

## 3 Proposed system

In the proposed audience participation game (APG), users are divided into two teams, Team P1 and Team P2. Each user supports their team's AI by pressing buttons, earning scores for their participation. The top-10 users with the highest scores are displayed on a leaderboard. The game offers both competitive and collaborative modes. In the competitive game, users are split into Team P1 and Team P2, while in the collaborative game, all users are on the same side (Team P1).

**Figure 1:**
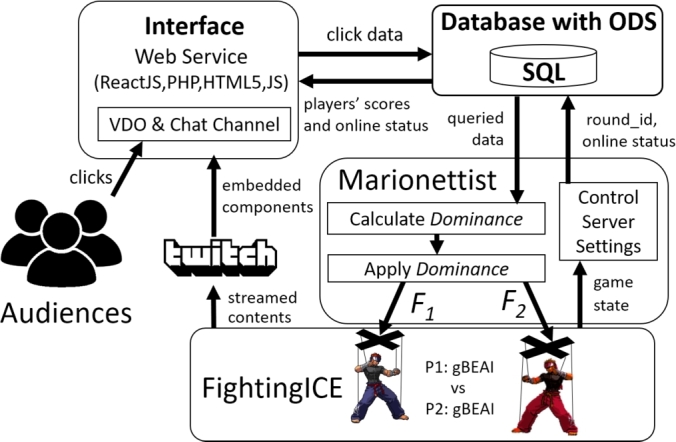
The architecture of the game system.

The architecture of the game system is depicted in Fig. 1. It consists of four main components, each with further details provided in subsequent subsections.

### 3.1 Interface

The interface of the APG is a web application hosted on a web server. Users are required to log in when they visit the index page. In the official version, users must have an account, which can be registered on the site using Firebase authentication. Email verification is required, but a Twitch account is not necessary. However, for beta tests, there is an alternative index page where users can enter the system by providing only a preferred display name without registration. Upon logging in, users select their team, either “Team P1” or “Team P2.”

**Figure 2:**
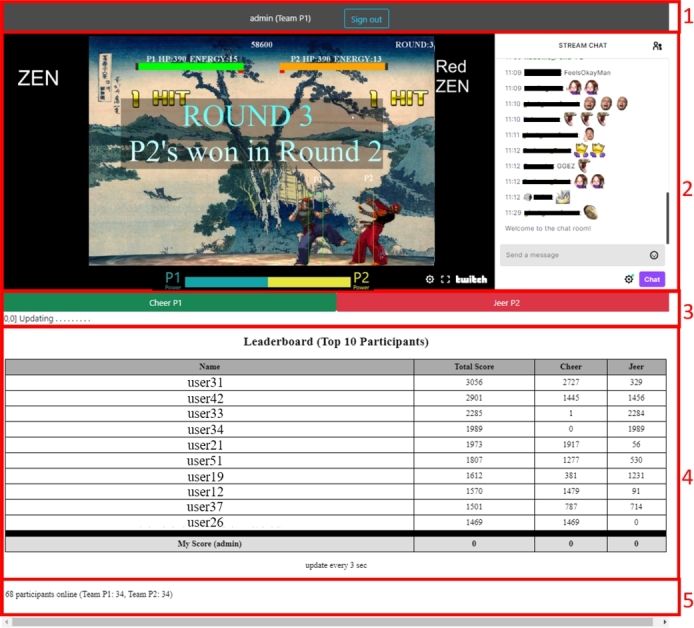
The screenshot of the system running on a web browser displays a vertically divided page with five components: (1) the display name and team of the current user, along with a log-out button, (2) a live-stream video and a chat area located on the right side of the page, (3) buttons for cheering and jeering, (4) a leaderboard showcasing the top-10 most active users, (5) the number of users currently online.

Fig. 2 illustrates the main page of the APG after logging in, divided into five components. Component 1 at the top displays the user's name and team, along with a logout button. Component 2 contains the embedded Twitch streaming content and chat area. The streaming content includes the game screen and additional text for character names and battle results. Component 3 presents two buttons: a green button for cheering (to strengthen the user's team AI) and a red button for jeering (to weaken the opponent AI). Component 4 displays a leaderboard, with the user's score added at the bottom. Component 5 indicates the number of users currently online, providing information about the team membership count.

The interface of the APG was developed using a combination of web programming languages. The client-side, including the login page and the container for all components shown in Fig. 1, was implemented using ReactJS. The content for Component 4 (leaderboard) and Component 5 (online user count) is updated every 3 and 10 seconds, respectively. This is achieved by the ReactJS page making requests to PHP scripts on the server-side. These PHP scripts retrieve data from an SQL database and render the HTML components accordingly.

### 3.2 Database with ODS

The system utilizes Structured Query Language (SQL) to store all interface usage data in a game database. This database serves as an operational data store (ODS) and contains tables for tracking user sessions and game rounds. Counters are implemented to facilitate the generation of leaderboards. The variables stored in the interface and gameplay controller are presented in Table 2. The interface employs Algorithm 1, Algorithm 2 for updating Component 4 and Component 5, respectively, as depicted in Fig. 2. The detailed database schema, SQL commands, and comprehensive versions of the algorithms can be accessed on osf.io.^6^

**Table 2:** Variables used in the game system.

*variablename*	definition
*name*	name of the user
*team*	team of the user
*c*1	the number of cheers for P1 in the buffer
*j*1	the number of jeers for P1 in the buffer
*c*2	the number of cheers for P2 in the buffer
*j*2	the number of jeers for P2 in the buffer
*o*	binary integer telling if the game is online
*s*	id of the current session (group of users)
*r*	id of the current game round

**Algorithm 1:**
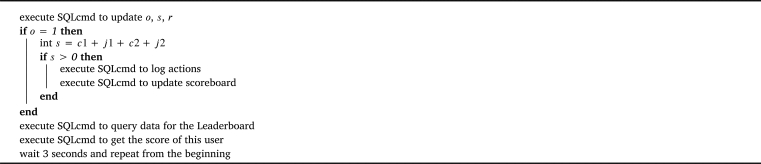
Interface Update: Leaderboard.

**Algorithm 2:**
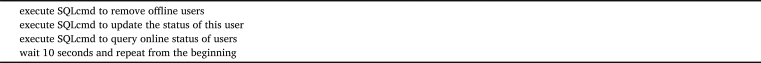
Interface Update: online status.

### 3.3 Marionettist (gameplay controller)

Marionettist is a component or sub-system responsible for controlling the strength of AI in the game and updating the game server status (i.e., online and round_id). As aforementioned, the game system uses two gBEAIs. The strength of each gBEAI is controlled by a parameter *F*. In Marionettist, a new parameter named “Dominance (*D*)” is introduced–inspired by a study on a dominance adjustment AI by Xu et al. [30]. This parameter is simply equal to $-F^{P1}$ and $-F^{P2}$ (cf. Eq. (1)), ensuring its range is [-1, 1]. Positive values indicate P1's dominance in the game, with a value of 1 corresponding to P1 winning by a 120 HP difference. Negative values indicate P2's dominance, while a value of 0 represents an equal match.

(1) $$D=-F^{P1}=F^{P2}$$

Eq. (1) can be broken down to Eq. (2), where $k^{P1}$ and $k^{P2}$ are the total number of clicks favoring P1 and P2 respectively. $k^{P1}$ can be either cheering P1 or jeering P2 (cf. Eq. (3)), while $k^{P2}$ can be either cheering P2 or jeering P1 (cf. Eq. (4)). These inputs are obtained by executing SQLcmd to obtain data for updating *D* every one second (cf. Algorithm 3).

(2) $$D=\frac{k^{P1}-k^{P2}}{k}=\frac{k^{P1}-k^{P2}}{k^{P1}+k^{P2}}$$

(3) $$k^{P1}=total\_cheer\_P1+total\_jeer\_P2$$

(4) $$k^{P2}=\left\{ \begin{array}{cc} total\_cheer\_P2+total\_jeer\_P1, & \text{Competitive}\\ x\cdot \mathrm{arg min}(t,\Delta ), & \text{Collaborative} \end{array}\right.$$

**Algorithm 3:**
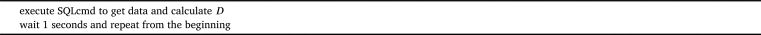
Game Update: adjust AIs' strength.

In the Collaborative Game mode, P2 receives no clicks of support from the users. Consequently, the parameter $k^{P2}$, which quantifies support for P2, follows a distinct calculation method. This approach is designed with the expectation that supporters of P1 need to maintain their previous level of activity, on average and adjusted for the number of players, to assist P1 in securing victory in the game.

Specifically, $k^{P2}$ is determined based on a parameter *x* (as indicated in Eq. (5)), representing the average number of clicks per user per second. In this equation, $k_{r-1}$ denotes the total number of clicks in the last round, and *n* represents the total number of users. The value of *x* is regularly updated at the beginning of each game round, utilizing data obtained from SQLcmd.

To calculate $k^{P2}$, the parameter *x* is multiplied by the elapsed game time in seconds since the round began (*t*). This calculation takes into account the support for P2 over time in the Collaborative Game mode.

(5) $$x=\frac{k_{r-1}\cdot n}{60\cdot n_{r-1}}$$

### 3.4 FightingICE and gBEAIs

In the modified version of FightingICE for the APG, no changes were made to the game itself, only to the AI component. The gBEAI (Believable Entertaining AI) was modified to receive a parameter indicating the targeted HP difference. Additionally, the gBEAI was modified to send game state data to the Marionettist, which helps notify when a game round begins and ends.

In terms of visual enhancements, texts displaying character names, round announcements, and previous round results were added to the game screen. These texts are exported from the AI as text files and read by OBS,^7^ an open-source software used for Twitch live streaming, to display them as part of the streaming content. Additionally, a bar with blue and yellow colors is provided by the Marionettist below the game screen to indicate which side is currently dominating the game.

## 4 Experiment and results: AI test

In a similar manner to our previous work [18], we conducted an evaluation of the performance of the proposed AI. Specifically, we focused on investigating the time it takes for two gBEAIs to adjust their strengths and converge on the targeted HP difference, while varying the value of the parameter *D*.

### 4.1 Apparatus

The tests were conducted using Fighting ver.4.5, with both sides using the “ZEN” character.^8^ The parameters of the skills for this character are publicly accessible. The MCTS parameters were set based on a previous study [10]. The tests were performed on Dell Computers with an Intel(R) Xeon(R) W-2135 CPU running at 3.70 GHz, 16 GB of RAM, and Windows 10 Pro for Workstations x64.

### 4.2 Settings and results

**Figure 3:**
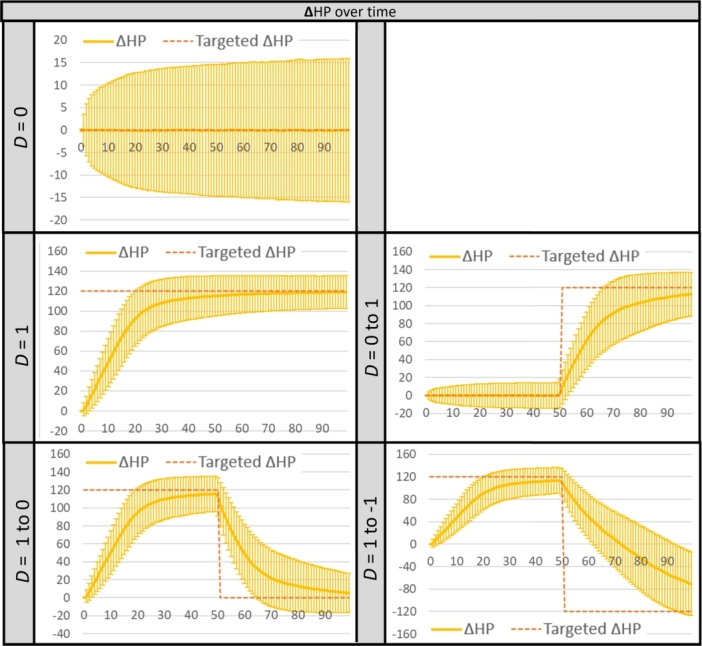
Game test results for different settings of *D*; each was from a ten thousand 1-min rounds. Δ*HP* represents a mean with error bars of the absolute HP difference between the two characters. The x-axis represents the time point (*t*), where *t* = 50 and 100 are the middle and the end of a 1-min round.

The tests involved 9 different settings of the dominance parameter *D*, each tested with various combinations of *D* values and a total of 50,000 rounds, except for setting $D_{1}$ which had 100,000 rounds. The tests were conducted in Time Mode, where each round had a fixed duration of 60 seconds, and the HP values started from 0 and could go into negative values. The results from the same pair of settings were combined, resulting in 5 settings of *D* with data from 100,000 rounds each. These results, displayed in Fig. 3, depict the HP difference (Δ*HP*) between Player 1 (P1) and Player 2 (P2), calculated by subtracting P2's HP from P1's HP.

The results showed that in most cases, the mean HP difference (Δ*HP*) reached around the targeted value during the middle and end of the game. The worst case occurred when *D* was set to 1 to 1, where there was a difference of about 200 units from the targeted value in the last 30 seconds of the game. Overall, more details and results about this AI test can be found on the OSF platform.^9^

## 5 Experiment and results: user experiment

We conducted a user experiment to evaluate the impact of different designs on user experience (UX) and activeness in our game. The experiment compared two designs for audience participation, namely “Competitive” and “Collaborative.” We also included a baseline condition called “Watch-only,” where participants watched the game on Twitch without interacting with the interface. The game mode used was HP Mode, where each character starts with 400 HP, and the round ends when one character's HP reaches 0 or when the timeout is reached. Both P1 and P2 used the ZEN character, with different character skins but the same skills.

### 5.1 Research questions

We are investigating areas that have not yet been well-studied, and this study is exploratory research.^10^ Six global research questions were formulated as follows:

1) Do APG and Non-APG affect UX differently? (Answered in Section 5.6.2)
2) Do Collaborative and Competitive games affect UX differently? (Answered in Section 5.6.2)
3) Do “Cheer” and “Jeer” affect UX differently? (Answered in Section 5.6.3)
4) Comparing collaborative and competitive games, do users cheer and jeer differently? (Answered in Section 5.6.3)
5) Are there any associations between activeness and UX? (Answered in Section 5.6.4)
6) Do users with different levels of familiarity with Twitch and fighting games perceive different UX? (Answered in Section 5.6.5)

In this study, we aim to provide comprehensive answers to the research questions by analyzing not only “yes” or “no” responses but also exploring the “how” and “why” aspects. User experience (UX) will be examined across four independent gaming aspects: Play Engrossment, Enjoyment, Personal Gratification, and Social Connectivity. The experimental results will generate hypotheses regarding the factors that potentially contribute to UX in each specific aspect. These hypotheses will serve as a basis for future experiments on games of various genres to further validate and confirm the findings. Following the approach of previous studies (e.g., [19]), we did not employ Bonferroni correction due to the exploratory nature of the study.

### 5.2 UX assessment

To assess the user experience (UX), the Game User Experience Satisfaction Scale (GUESS) developed by Phan et al. [20] was utilized. GUESS evaluates UX based on various factors, with each factor representing a specific gaming aspect. In this study, four factors that were deemed applicable to the research context were chosen: Play Engrossment, Enjoyment, Personal Gratification, and Social Connectivity. To measure these factors, three questions were selected for each, adapting them slightly to align with the characteristics of our game system.

In contrast to the original GUESS questionnaire, which uses a “ranking (scaling)” scheme for self-reporting, our modified GUESS utilizes a “pairwise preference” approach. In our 4-AFC (4-alternative forced choice) GUESS questionnaire (cf. Table 3), users are presented with two different experimental conditions and asked to compare them. This modification was inspired by recent research in game studies, such as studies by Yannakakis et al. (e.g., [31], [32]), which have shown the benefits of using pairwise preferences. This approach minimizes assumptions about subjective constructs, reduces inter-personal differences, and eliminates artifacts related to ranking/scaling. The modified GUESS questions were adapted based on templates from previous studies conducted by Yannakakis et al. [32] and Burelli et al. [3].

**Table 3:** 4-AFC version of the Game User Experience Satisfaction Scale (GUESS).

Factor	Questions
Play Engrossment	1. In which game you felt more detached from the outside world while participating.
(**Eg**)	2. In which game you cared less to check events that were happening in the real world during the game.
	3. In which game you were getting less tired while participating.

Enjoyment	1. In which game you thought was more fun.
(**Ej**)	2. In which game you more enjoyed participating
	3. In which game you felt more bored while participating.

Personal	1. In which game you were more in suspense about whether your player would succeed.
Gratification (**PG**)	2. In which game you felt more successful when your player overcame the obstacles.
	3. In which game you wanted more to “cheer to help my player” / “jeer to hurt the opponent” as well as possible during the game.

Social	1. In which game you found it better supported social interaction between audiences.
Connectivity (**SC**)	2. In which game you liked more to participate with other audiences.
	3. In which game you were more able to participate with other audiences if you chose.

Four answer choices: (1) Game 1, (2) Game 2, (3) Both Equally, (4) Neither.

### 5.3 Candidates and participants

Initially, we began with a pool of 161 potential candidates from two separate class sessions, consisting of 80 participants in one session and 81 in the other. Subsequently, after accounting for absences and those who declined to participate, we had 82 participants actively engage in the AP. From this group, some individuals either failed to complete the required forms and questionnaires, or experienced issues with data submission through the online Survey Monkey platform. As a result, a total of 75 participants successfully completed the questionnaire. However, data from one participant had to be excluded because they provided identical responses to all questions and did not actively participate. Therefore, when excluding those who did not actively engage during the experiment, our final sample consisted of 74 participants, distributed across both class sessions (39 in one session and 35 in the other). Among those participants who disclosed their ages (62 out of 74), the mean age was 21.68 ± 5.83. In terms of gender distribution, there were 43 males, 3 females, and the remaining participants identified as non-binary or chose not to specify their gender.

### 5.4 Procedures

The experiment was conducted remotely during the COVID-19 pandemic via ZOOM cloud meeting. Procedures were as the followings.

#### 5.4.1 Introduction and the informed consent

The experiment began with a 10-minute introduction where participants were provided with detailed information about the research study. During the first 5 minutes, participants were given a link to submit an online informed consent form, and the items in the form were explained to them. The remaining 5 minutes were provided for participants to read the informed consent form silently before submitting it.

Candidates were informed about the purpose of the research study, how to play FightingICE, the removal of personal information, and the voluntary nature of participation without any disadvantageous consequences for choosing not to participate. They were informed that we were testing one of our designed APG interfaces but were not provided with specific details about the interfaces used by other groups (i.e., the comparison between Competitive and Collaborative was not disclosed). The informed consent form was created based on the research ethics guidelines at the authors' universities, and according to these guidelines, the research did not require ethics approval. The consent form and the 4-AFC GUESS questionnaire were provided in both English and Thai translations, and the explanations were given in Thai.

A total of 119 consent forms were submitted for the study, with 82 participants agreeing to participate. Furthermore, in accordance with the guidelines at the authors' university, the research does not require ethics approval, and all human subject research procedures and protocols are exempt from review board approval.

### 5.5 Experiment and the questionnaire

Both groups initially started with a Watch-only game, consisting of three 1-minute rounds. Afterward, there was a 3-minute break during which participants were provided with details about the next game they would participate in (either Competitive or Collaborative). Following the game session, participants proceeded to complete the questionnaire. The expected duration for the entire experiment, including the games and the questionnaire, was 19 minutes, with an additional 5 minutes allocated for the questionnaire.

### 5.6 Results

#### 5.6.1 Frequencies of responses

**Figure 4:**
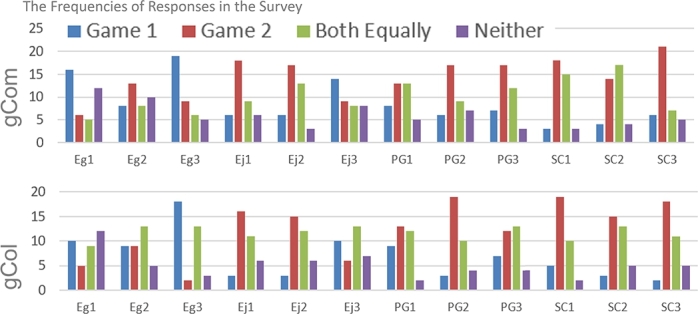
Frequencies of responses from the GUESS questionnaire (raw results).

A total of 75 participants completed the questionnaire, but data from one participant were excluded as they provided the same answer to all questions and did not actively participate. Therefore, data from 74 participants were used for analysis. The frequency histogram of the GUESS questionnaire responses is presented in Fig. 4. Group I (gCom) refers to participants who experienced the Watch-only game followed by the Competitive Game, while Group II (gCol) refers to participants who experienced the Watch-only game followed by the Collaborative Game.

The pre-processing step involved combining the answers from the three questions into a single “final answer” to indicate each participant's preference for a specific UX factor. This was done by taking the majority answer from the three questions. In cases where there was no clear majority, the final answer was labeled as “unclear” (labeled as “0”). For the third question assessing Enjoyment, which was a negative statement, answers 1 and 2, as well as 3 and 4, were switched before determining the final answer.

**Figure 5:**
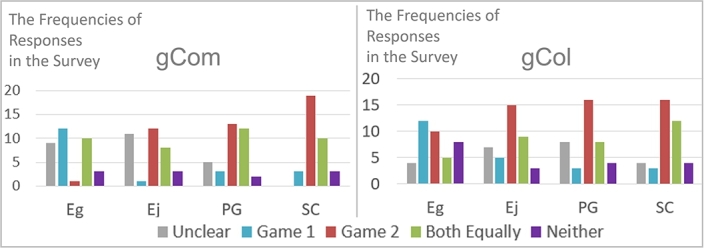
Frequencies of final answers.

The frequency histogram of the final answers is displayed in Fig. 5, and the corresponding values are provided in Table 4.

**Table 4:** Preferences of participants based on four UX factors.

	Answer*	n̂**	Eg	Ej	PG	SC
Group 1 (gCom)	0	14.6	4	7	8	4
Competitive	1	6.1	12	5	3	3
(*N* = 39)	2	6.1	10	15	16	16
	3	6.1	5	9	8	12
	4	6.1	8	3	4	4

Group 2 (gCol)	0	13.1	9	11	5	0
Collaborative	1	5.5	12	1	3	3
(*N* = 35)	2	5.5	1	12	13	19
	3	5.5	10	8	12	10
	4	5.5	3	3	2	3

*Answer: (0) Unclear, (1) Prefer Game 1, (2) Prefer Game 2, (3) Prefer Both Equally, (4) Prefer Neither.

**n̂: expected frequency assuming that the result was obtained by chance.

#### 5.6.2 Difference between experimental conditions

The analysis began with a chi-square goodness-of-fit test in SPSS Statistics to determine if the obtained results differed significantly from chance. There were a total of 64 possible combinations of answers for each factor, and if the results were obtained by chance, the expected proportions would be 24 for “Answer 0” and 10 each for the remaining answers. The test results indicated that the frequencies of all UX factors in both groups were significantly different from the expected values when considering all types of answers.

The next step examined whether the two different game conditions, Non-APG and APG, had different effects on UX by comparing Answer 1 and 2 using a binomial test. The expected ratio was 1:1. The results, shown in Table 5, revealed differences between the two conditions for all factors in both groups, except for Engrossment in Group 1.

**Table 5:** P-values from statistical tests (goodness-of-fit).

	Group	Eg	Ej	PG	SC
Chi, goodness-of-fit,	gCom	**.002**	**.000**	**.000**	**.000**
comparing all choices	gCol	*	**.001**	**.007**	**.000**

Binomial test,	gCom	.835	**.041**	**.004**	**.004**
comparing Game 1 and 2	gCol	**.003**	**.003**	**.021**	**.001**

* *p*-value cannot be obtained since the frequency of “Answer 0” is zero (the expected frequency is 13.1); the difference was explicit on observation.

Considering the frequencies in Fig. 5, it can be concluded that APG (either Competitive or Collaborative) was more preferred than Non-APG in terms of Enjoyment, Personal Gratification, and Social Connectivity. However, participants perceived significantly lower Engrossment in Collaborative APGs compared to Non-APG. In Competitive APGs, a similar perception was found, but the difference was smaller and not statistically significant (12 for Non-APG vs. 10 for Competitive APG).

We also ran a chi-square test for association to find correlations between group of participants and UX factors. A significant difference was found (cg. Table 6) on Engrossment with a *p*-value of .011, which confirms the difference found above.

**Table 6:** P-values from statistical tests (associations).

	Group	Eg	Ej	PG	SC
Group	-	**.011**	.442	.688	.357

Type of Audience	Both	.283	.899	.468	.254
Type of Audience	gCom	.313	.546	.734	.476
Type of Audience	gCol	.757	.116	.713	.084

Top10	Both	.692	.356	.370	.335
Top10	gCom	.652	.711	**.038**	.110
Top10	gCol	.636	.545	.228	.989
Rank Group	Both	.741	.241	.177	.383
Rank Group	gCom	.701	.344	.477	.874
Rank Group	gCol	.396	.385	.078	.068

Familiarity to Twitch	Both	.675	.060	.663	.352
Familiarity to Twitch	gCom	.678	.384	.480	**.047**
Familiarity to Twitch	gCol	.564	.377	.735	.511
Familiarity to FTG	Both	**.046**	.089	.862	.829
Familiarity to FTG	gCom	.639	.501	.837	.733
Familiarity to FTG	gCol	**.045**	.433	.505	.622

#### 5.6.3 Association - audience types

Participants were categorized into three types of audiences based on their click logs: “Cheer Audience (Cheerer),” “Jeer Audience (Jeerer),” and “Neutral Audience (Neutral).” Classification was done using the ratio $\frac{Cheer}{Cheer+Jeer}$, where values greater than 0.666 indicated Cheerer, values less than 0.333 indicated Jeerer, and values in between indicated Neutral.

Chi-square tests for association were conducted to examine the relationship between audience type and UX factors, as well as between audience type and experimental group. The results, shown in Table 6, indicated no significant associations between audience type and UX factors or between audience type and experimental group. However, in terms of the numbers, Group 1 had 23 Cheerers, 11 Jeerers, and 5 Neutrals, while Group 2 had 49 Cheerers, 16 Jeerers, and 9 Neutrals. In terms of percentages, Jeerers accounted for 28.20% of participants in the Competitive Game, compared to 14.28% in the Collaborative Game.

Based on these findings, it can be concluded that the presence of Cheer or Jeer did not significantly affect UX differently. However, participants tended to engage in more Jeering behavior in Collaborative games where the opposing side did not involve human players.

#### 5.6.4 Association - activeness

Activeness, which represents the level of participation, was measured and discretized to create categorical variables for association tests. Participants in each group were ranked based on their user scores, which corresponded to the total number of clicks. Discretization was performed, resulting in two categorical variables.

The first variable, “Top10,” determined whether a participant ranked among the top ten, whose scores were displayed on the leaderboard at the end of the experiment. The second variable, “Rank Group,” divided participants in each experimental group into five equal groups based on their rankings, with Group 1 being the most active and Group 5 being the least active.

Using chi-square tests for association (see Table 6), it was found that top-10 and non-top-10 participants in Group 1 perceived Personal Gratification differently. Frequencies revealed that none of the top-10 participants preferred Game 1, indicating that Competitive APG enhanced Personal Gratification.

Associations between Top10 (or Rank Group) and Type of Audience were also analyzed, but no significant association was found. This suggests that all types of audience were present across all levels of activeness, indicating that all types of audience were equally active in the experiment.

#### 5.6.5 Association - familiarity

**Figure 6:**
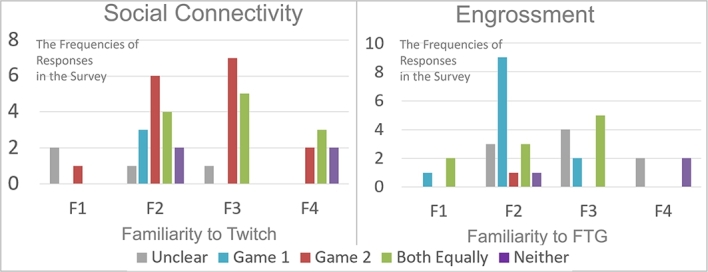
Frequencies based on familiarity.
• In Group 2, familiarity with fighting games affected the perception of Engrossment. The frequencies indicated that as the level of familiarity increased, there was a decrease in preferences towards Non-APG. Participants familiar with fighting games tended to have unclear preferences or perceive no engrossment from both games.

Two important findings were obtained regarding familiarity with Twitch and fighting games (FTG) in relation to the perception of certain factors:

• In Group 1, familiarity with Twitch influenced the perception of Social Connectivity. The frequencies showed that as the level of familiarity increased, there was a decrease in unclear preferences and preferences towards Non-APG. The left image in Fig. 6 shows frequencies over this association.

These findings suggest that familiarity with Twitch and fighting games can have an impact on the perception of specific factors in the user experience.

#### 5.6.6 Other findings

The analysis of questionnaires, game logs, and open-ended responses, after filtering out positive and irrelevant comments, revealed that in the competitive game, both teams (P1 and P2) were evenly matched, with Team P1 narrowly winning all three rounds. However, the game's outcome (win/lose) did not significantly impact participants' user experience (UX).

Regarding technical aspects, there was no observed lag in the APG with 82 participants, and approximately 8941 cheer/jeer messages were generated per game round (lasting 1 minute). However, it's worth noting that a buffer is used on the client side of the application. This means that the total number of clicks for cheer and jeer buttons is counted and sent to the server every second, rather than immediately upon clicking. Additionally, the calculations for adjusting the Dominance parameter are performed every second on the client side, while the leaderboard is updated every 3 seconds. Therefore, the system would not encounter issues due to users clicking too frequently.

One issue we encountered was that around a quarter of the participants (18 individuals) expressed concerns about the potential use of macros or auto clickers, emphasizing the importance of implementing a cooldown period between clicks to maintain fairness and enjoyment for all users.

Additionally, one concern arose regarding the current social facilitation mechanism in the game, which grants advantages to teams with more audience support, potentially compromising gameplay balance. While this design suits scenarios involving “Player with audiences vs. AI,” it requires adjustments to address the impact of uneven audience numbers in “AI with audiences vs. AI with audiences” gameplay. Furthermore, it is not currently applicable to “Player with audiences vs. Player with audiences” without modifications, as the concept revolves around adjusting the AI's strength. This suggests that balancing the two teams is one of the issues stemming from the current design, and it's important to note that even when the number of team members is even, it doesn't guarantee equal activeness of supporters between the two teams.

Among the limitations and issues identified, some audience members reported that their attention was divided between watching the gameplay and clicking the cheer/jeer buttons, which may affect their overall experience.

## 6 Conclusion and future works

This paper presented a novel audience participation fighting game that merged elements from FightingICE and Twitch. The system's design and architecture were thoroughly explained. The game featured two gameplay modes: collaborative and competitive, with two interaction options: cheering and jeering. To assess the impact of these gameplay and interaction types on user experience (UX) and participant activeness, a comparative analysis was conducted. The system underwent AI tests, and a user experiment, involving 82 participants, was performed to gather valuable insights into the effects of these factors on audience experience.

This study, based on the analysis of valid data from 74 participants, investigated the influence of various factors on user experience (UX) in Audience Participation Games (APGs). The results revealed that game conditions, including the presence of APG and the type of game (Competitive or Collaborative), had significant effects on user preferences related to Enjoyment, Personal Gratification, and Social Connectivity. Notably, Collaborative APGs exhibited lower levels of Engrossment compared to Non-APGs. The study also raised concerns about issues like cheating and potential device-related disparities, underscoring the need for effective fairness mechanisms in APGs. Furthermore, areas for future research, including audience demographics and game introductions, were identified. In summary, this research provides valuable insights into the nuanced dynamics of UX in APGs and underscores the importance of addressing specific challenges to enhance the overall gaming experience.

The experiment's findings have generated a set of hypotheses that lay the groundwork for future research into various aspects of Audience Participation Games (APGs). These hypotheses include: [H1] The impact of audience participation on Enjoyment, Personal Gratification, and Social Connectivity, with potential decreases in Engrossment in collaborative settings. [H2] Differential effects on Engrossment in competitive and collaborative games. [H3] The comparative influence of Cheering and Jeering on user experience. [H4] The tendency of audiences to jeer more in competitive games, with a note on the statistical significance. [H5] High perceived Personal Gratification among top players featured on leaderboards in competitive environments, and the potential unreliability of click-based activeness assessment. [H6] The influence of familiarity with Twitch and game genre on specific aspects of user experience. These hypotheses provide a solid foundation for further exploration of game genres, the impact of competitive versus collaborative gameplay, audience interactions, and player engagement within the realm of APG environments.

In future research, it is essential to tackle the issue of varying audience sizes on different teams, particularly when dealing with audience participation games (APGs). This entails developing strategies to handle scenarios in which team audience sizes are uneven, ensuring that competition remains enjoyable. Equally important is the implementation of mechanisms to prevent cheating and maintain fair play. Furthermore, a significant challenge lies in enhancing the adjustment of AI difficulty to provide a balanced and captivating gameplay experience. It's also imperative to develop mechanisms to prevent cheating and ensure fair play.

There is an ongoing challenge in improving the fine-tuning of AI strength to maintain a balanced and engaging gameplay experience. Researchers should also explore the adaptability of the proposed system architecture and interaction designs, centered around dynamic difficulty adjustment using the social facilitation concept, to a wide range of game genres, expanding the study's applicability beyond the specific genre examined in this research. It's worth noting that while the proposed parameter, Dominance (D), as shown in Eq. (2), can be extended to other games, the calculation of $k^{P1}$ and $k^{P2}$ may need to be adjusted to align with the characteristics of each specific game. Investigating the user experience of human players in Human vs AI scenarios, as previously conducted in traditional non-APG games (e.g., [1], [11], [13]), would be valuable. These future research directions aim to address challenges and advance the understanding of UX in APGs, ultimately contributing to the development of more engaging and enjoyable gaming experiences for both audiences and players.

## CRediT authorship contribution statement

**Pujana Paliyawan:** Writing – review & editing, Writing – original draft, Visualization, Validation, Software, Resources, Project administration, Methodology, Investigation, Formal analysis, Data curation, Conceptualization. **Ruck Thawonmas:** Writing – review & editing, Supervision, Resources, Project administration, Funding acquisition. **Kingkarn Sookhanaphibarn:** Writing – review & editing, Validation, Resources. **Worawat Choensawat:** Writing – review & editing, Validation, Resources.

## Declaration of Competing Interest

The authors declare the following financial interests/personal relationships which may be considered as potential competing interests:

We don't get any funding. But the co-author, Ruck Thawonmas, is an associate editor for the journal. The papers accepted with him as a co-author, regardless of their subject areas, will be exempted from publication fees.

## Data Availability

To promote reproducibility, we have provided supplementary information and research-related files, including the system architecture and experimental materials, on the Open Science Framework (OSF) platform (https://osf.io/vefrd/).

## Appendix A Supplementary material

MMC 1 The 4-AFC version of the Game User Experience Satisfaction Scale (GUESS), the online questionnaire utilized in the experiment. Printed from Survey Monkey. MMC 1

MMC 2 The Consent Form employed in the experiment. Printed from Survey Monkey. MMC 2

MMC 3 Experiment data comprising GUESS survey responses from participants in Group 1. MMC 3

MMC 4 Experiment data comprising GUESS survey responses from participants in Group 2. MMC 4

MMC 5 Link to the repository containing the source code and data associated with this project. MMC 5

## Footnotes

1 https://www.businessofapps.com/data/twitch-statistics/.

2 www.who.int/campaigns/connecting-the-world-to-combat-coronavirus/healthyathome/healthyathome---mental-health.

3 https://steamcommunity.com/app/405980.

4 https://www.ice.ci.ritsumei.ac.jp/~ftgaic/index-R.html.

5 http://www.ice.ci.ritsumei.ac.jp/%7eftgaic/index-2h.html.

6 https://osf.io/gpb7w/wiki/1.%20Database/.

7 an open source software used for Twitch live streaming.

8 ZEN Skill Table: https://www.ice.ci.ritsumei.ac.jp/~ftgaic/index-2a.html.

9 https://osf.io/6x2ep/.

10 https://researcher-help.prolific.co/hc/en-gb/articles/360009500513-Am-I-doing-exploratory-or-confirmatory-research-Why-does-it-matter-.
