# Supplementary material for: Audience participation fighting game: Exploring social facilitation for an enhanced APG experience
Source: Heliyon. 2024 Jan 2;10(2):e23967. doi: 10.1016/j.heliyon.2023.e23967 (PMC10826610; doi:10.1016/j.heliyon.2023.e23967)
Supplement: MMC 1 — The 4-AFC version of the Game User Experience Satisfaction Scale (GUESS), the online questionnaire utilized in the experiment. Printed from Survey Monkey. [file mmc1.pdf]

Audience Participation FIghtingICE - 4AFC-GUESS EN TH [BU]

* 1. Display name in the game system | ชื่อที่ใช้ในระบบ

2. Age | อายุ

3. Gender | เพศ

4. E-mail | อีเมล

* 5. Your level of familiarity with Twitch | ความคุ้นเคยกับ Twitch

- Not familiar at all | ไม่คุ้นเคยเลย
- Slightly familiar (have used it but don’t know it much) | คุ้นเคยเล็กน้อย (เคยใช้บ้าง แต่ไม่มากนัก)
- Somewhat familiar | ค่อนข้างคุ้นเคย
- Extremely familiar | คุ้นเคยเป็นอย่างดี

* 6. Your level of familiarity with fighting games | ความคุ้นเคยกับเกมต่อสู้

- Not familiar at all (have never played fighting games) | ไม่คุ้นเคยเลย (ไม่เคยเล่น)
- Slightly familiar (have played fighting games, but don’t know much about them) | คุ้นเคยเล็กน้อย (เคยเล่นบ้างแต่ไม่มากนัก)
- Somewhat familiar (I have a broad understanding of what fighting games are) | ค่อนข้างคุ้นเคย (รู้จักอย่างกว้างๆ)
- Extremely familiar (I have extensive knowledge on fighting games) | ค่อนข้างคุ้นเคยเป็นอย่างดี (มีความรู้เกี่ยวกับเกมต่อสู้)

7. Your Team

* 8. In which game you felt more detached from the outside world while participating.
เกมไหนที่คุณรู้สึกโดดเดี่ยวมากกว่าจากโลกภายนอก ในขณะที่มีส่วนร่วม

Game1<br>เกม1	Game2<br>เกม2	Both Equally<br>ทั้งสองเกมเท่าๆกัน	Neither<br>ไม่ใช่ทั้งสองเกม
◯	◯	◯	◯

* 9. In which game you cared less to check events that were happening in the real world during the game.
เกมไหนที่คุณสนใจน้อยกว่าที่จะตรวจสอบเหตุการณ์ที่เกิดขึ้นในโลกแห่งความจริง ระหว่างที่มีส่วนร่วม

Game1<br>เกม1	Game2<br>เกม2	Both Equally<br>ทั้งสองเกมเท่าๆกัน	Neither<br>ไม่ใช่ทั้งสองเกม
◯	◯	◯	◯

* 10. In which game you were getting less tired while participating.
เกมไหนที่คุณรู้สึกเหนื่อยน้อยกว่า ในขณะที่มีส่วนร่วม

Game1<br>เกม1	Game2<br>เกม2	Both Equally<br>ทั้งสองเกมเท่าๆกัน	Neither<br>ไม่ใช่ทั้งสองเกม
◯	◯	◯	◯

* 11. In which game you thought was more fun.
เกมไหนที่คุณคิดว่าสนุกมากกว่า

Game1<br>เกม1	Game2<br>เกม2	Both Equally<br>ทั้งสองเกมเท่าๆกัน	Neither<br>ไม่ใช่ทั้งสองเกม
◯	◯	◯	◯

* 12. In which game you more enjoyed participating.
เกมไหนที่คุณสนุกมากกว่ากับการมีส่วนร่วม

Game1<br>เกม1	Game2<br>เกม2	Both Equally<br>ทั้งสองเกมเท่าๆกัน	Neither<br>ไม่ใช่ทั้งสองเกม
◯	◯	◯	◯

* 13. In which game you felt more bored while participating.
เกมไหนที่คุณรู้สึกเบื่อมากกว่า ในขณะที่มีส่วนร่วม

Game1<br>เกม1	Game2<br>เกม2	Both Equally<br>ทั้งสองเกมเท่าๆกัน	Neither<br>ไม่ใช่ทั้งสองเกม
◯	◯	◯	◯

* 14. In which game you were more in suspense about whether your player would succeed.
เกมไหนที่คุณสงสัยมากกว่าว่าผู้เล่นฝ่ายคุณจะประสบความสำเร็จในเกมหรือไม่

Game1<br>เกม1	Game2<br>เกม2	Both Equally<br>ทั้งสองเกมเท่าๆกัน	Neither<br>ไม่ใช่ทั้งสองเกม
○	○	○	○

* 15. In which game you felt more successful when your player overcame the obstacles.
เกมไหนที่คุณรู้สึกประสบความสำเร็จมากกว่าเมื่อผู้เล่นฝ่ายคุณเอาชนะอุปสรรคในเกมได้

Game1<br>เกม1	Game2<br>เกม2	Both Equally<br>ทั้งสองเกมเท่าๆกัน	Neither<br>ไม่ใช่ทั้งสองเกม
○	○	○	○

* 16. In which game you wanted more to "cheer to help my player" / "jeer to hurt the opponent" as well as possible during the game.
เกมไหนที่คุณอยากมากกว่าที่จะ "เชียร์เพื่อช่วยผู้เล่นฝ่ายคุณ" / "โห่เพื่อทำร้ายผู้เล่นฝ่ายตรงข้าม" ให้ดีที่สุดในระหว่างเกม

Game1<br>เกม1	Game2<br>เกม2	Both Equally<br>ทั้งสองเกมเท่าๆกัน	Neither<br>ไม่ใช่ทั้งสองเกม
○	○	○	○

* 17. In which game you found it better supported social interaction between audiences.
เกมไหนที่คุณพบว่ารองรับได้ดีกว่ากับการโต้ตอบทางสังคมระหว่างผู้เข้าร่วม

Game1<br>เกม1	Game2<br>เกม2	Both Equally<br>ทั้งสองเกมเท่าๆกัน	Neither<br>ไม่ใช่ทั้งสองเกม
○	○	○	○

* 18. In which game you liked more to participate with other audiences.
เกมไหนที่คุณชอบมากกว่าที่จะเล่นกับผู้เข้าร่วมคนอื่น ๆ

Game1<br>เกม1	Game2<br>เกม2	Both Equally<br>ทั้งสองเกมเท่าๆกัน	Neither<br>ไม่ใช่ทั้งสองเกม
○	○	○	○

* 19. In which game you were more able to participate with other audiences if you chose.
เกมไหนที่คุณมีความสามารถมากกว่าที่จะมีส่วนร่วมกับผู้ชมคนอื่นๆ หากคุณเลือก

Game1<br>เกม1	Game2<br>เกม2	Both Equally<br>ทั้งสองเกมเท่าๆกัน	Neither<br>ไม่ใช่ทั้งสองเกม
○	○	○	○

Audience Participation FIghtingICE - 4AFC-GUESS EN TH [BU]

Your opinion on the game

We appreciate your help.

20. Please write one good point about this game system (FightingICE + APG Interface)
กรุณาเขียนจุดที่ดีอย่างหนึ่งเกี่ยวกับระบบเกมนี้ (FightingICE + APG Interface)

21. Please write one bad point about this game system (FightingICE + APG Interface)
กรุณาเขียนจุดที่ไม่ดีอย่างหนึ่งเกี่ยวกับระบบเกมนี้ (FightingICE + APG Interface)

22. Your comments on how to improve the game (if any)
ข้อเสนอแนะเพิ่มเติมสำหรับปรับปรุงระบบ (ถ้ามี)
