# Supplementary material for: Audience participation fighting game: Exploring social facilitation for an enhanced APG experience
Source: Heliyon. 2024 Jan 2;10(2):e23967. doi: 10.1016/j.heliyon.2023.e23967 (PMC10826610; doi:10.1016/j.heliyon.2023.e23967)
Supplement: MMC 2 — The Consent Form employed in the experiment. Printed from Survey Monkey. [file mmc2.pdf]

CONSENT TO PARTICIPATE IN RESEARCH - Audience Participation FIghtingICE | ความยินยอมเข้าร่วมในการวิจัยหัวข้อ Audience Participation FIghtingICE (EN TH) [BU Group1]

** ภาษาไทยอยู่ด้านล่างจากภาษาอังกฤษ**

We will carry out this research as follows. If you understand the purpose of the research as well as the content of the research, and you can participate in this research, please sign the consent form. Even if you do not participate in the research or do decide to participate but decline in the middle, you will not suffer any disadvantage. We would be pleased if you could attend the research at your will.
เราจะดำเนินการวิจัยนี้ดังต่อไปนี้ หากคุณเข้าใจวัตถุประสงค์ของการวิจัยเช่นเดียวกับเนื้อหาของการวิจัย
และคุณสามารถเข้าร่วมในการวิจัยนี้ได้ โปรดลงนามในแบบฟอร์มยินยอม
แม้ว่าคุณจะไม่ได้เข้าร่วมในการวิจัยหรือตัดสินใจที่จะเข้าร่วมแต่ปฏิเสธกลางคัน คุณจะไม่ได้รับผลกระทบเชิงลบใดๆ
เรายินดีเป็นอย่างยิ่งหากคุณสามารถเข้าร่วมการวิจัยได้ด้วยความสมัครใจ

1.Significance and purpose of research
In this study, we present an interface for participating in Audience Participation Fighting Game and study its effects on user experience.
1.ความสำคัญและวัตถุประสงค์ของการวิจัย
ในการศึกษานี้ เรานำเสนออินเทอร์เฟซสำหรับการเข้าร่วมใน Audience Participation Fighting Game (เกมต่อสู้ที่ให้ผู้ชมมีส่วนร่วม)
และศึกษาผลกระทบต่อประสบการณ์ของผู้ใช้

2.Participation period and specific method
This experiment will take about 24 minutes. The experiment time and its contents are as follows.
2.1. Introduction and Consent Form (~10 minutes)
2.2. Game 1 (3 rounds, 3 minutes in total)
2.3. Break (~3 minutes)
2.4. Game 2 (3 rounds, 3 minutes in total)
2.5. Questionnaire (~5 minutes)
2. ระยะเวลาการเข้าร่วมและวิธีการ
การทดลองนี้จะใช้เวลาประมาณ 24 นาที เวลาทดลองและเนื้อหามีดังนี้
2.1. บทนำและเอกสารแสดงความยินยอม (~10 นาที)
2.2. เกมที่ 1 (3 round รวมแล้ว 3 นาที)
2.3. พัก (~3 นาที)
2.4. เกมที่ 2 (3 round รวมแล้ว 3 นาที)
2.5. แบบสอบถาม (~5 นาที)

3.Reasons for being selected as a research subject
This research targets people who had experience in a fighting game called FightingICE. This experiment requests all participants to play this game on a website.
3.เหตุผลถูกที่เลือกให้เข้าร่วมการวิจัย
งานวิจัยนี้กำหนดเป้าหมายผู้ที่เคยมีประสบการณ์ในเกมต่อสู้ที่ชื่อ FightingICE การทดลองนี้ขอให้ผู้เข้าร่วมทั้งหมดเล่นเกมนี้บนเว็บไซต์

4.Participation in the study is voluntary and participants have the right to decline or withdraw from the study
We hope you understand the purpose of the research and participate, but please decide by yourself whether to participate. You can also ask for an explanation about the purpose of the research. Even if you do not participate in the research or do decide to participate but decline in the middle, you will not suffer disadvantageous countermeasures. If you quit participation, please let us know what you would like us to do with the collected data so far: we will handle the data accordingly whether you would like to have it analyzed or abandoned.
4.การมีส่วนร่วมในการศึกษาวิจัยเป็นไปโดยสมัครใจและผู้เข้าร่วมมีสิทธิ์ปฏิเสธหรือถอนตัวจากการศึกษา
เราหวังว่าคุณจะเข้าใจวัตถุประสงค์ของการวิจัยและเข้าร่วม แต่โปรดตัดสินใจด้วยตัวเองว่าจะเข้าร่วมหรือไม่
คุณสามารถขอคำอธิบายเกี่ยวกับวัตถุประสงค์ของการวิจัยได้เช่นกัน แม้ว่าคุณจะไม่ได้เข้าร่วมในการวิจัย
หรือตัดสินใจที่จะเข้าร่วมแตถอนตัวภายหลัง คุณจะไม่ได้รับผลกระทบทางลบ หากคุณออกจากการเข้าร่วม
โปรดแจ้งให้เราทราบว่าคุณต้องการให้เราดำเนินการอย่างไรกับข้อมูลที่รวบรวมมา: เราจะจัดการข้อมูลตามนั้น
ไม่ว่าคุณต้องการให้วิเคราะห์หรือถอนออก

5.Participants will not receive any disadvantageous response by not agreeing to participate in the research or withdrawing from the research
You will not receive any disadvantageous response by not agreeing to participate in the research or withdrawing from the research.
5.ผู้เข้าร่วมจะไม่ได้รับผลกระทบเชิงลบใดๆ จากการไม่ตกลงที่จะเข้าร่วมในการวิจัยหรือถอนตัวจากการวิจัย
คุณจะไม่ได้รับผลกระทบเชิงลบใดๆ จากการไม่ตกลงที่จะเข้าร่วมในการวิจัยหรือถอนตัวจากการวิจัย

6.Expected benefits of participating in research
Participating in this research will not directly benefit you. But data collected from you will help us implement and develop audience participation games and comment generation that allows players to enjoy games more; We are expected to benefit the game industries in the future.
6.ผลประโยชน์ที่คาดหวังจากการเข้าร่วมการวิจัย
การเข้าร่วมในการวิจัยนี้จะไม่เกิดประโยชน์โดยตรงต่อคุณ
แต่ข้อมูลที่รวบรวมจากคุณจะช่วยให้เราสามารถนำไปใช้และพัฒนาเกมการมีส่วนร่วมของผู้ชมและการสร้างความคิดเห็นที่ช่วยให้ผู้เล่นสนุกกับเกมได้มากขึ้น เราคาดว่าจะเป็นประโยชน์ต่ออุตสาหกรรมเกมในอนาคต

7.Predicted risks, dangers, discomforts and effects on the body and mind
We have set the experiment time taking into account the fatigue of participants into consideration. If you normally play the game the following cases will not occur: (1) your eye strain occurs due to continuing to concentrate on the game screen or (2) your fingers get hurt.
7.พยากรณ์ความเสี่ยง อันตราย ความไม่สบาย และผลกระทบต่อร่างกายและจิตใจ
เราได้กำหนดเวลาการทดสอบโดยคำนึงถึงความเหนื่อยล้าของผู้เข้าร่วมด้วย หากคุณเล่นเกมตามปกติ ไม่น่าจะเกิดกรณีต่อไปนี้: (1) อาการปวดตาของคุณเกิดจากการจดจ่อกับหน้าจอเกมต่อไป หรือ (2) นิ้วของคุณได้รับบาดเจ็บ

8. Publication of results, possibility of feedback (Including all cases regardless of individual identifiable/unidentifiable at the time of research presentation)
The results of this research will be announced as academic achievements, but personal information, such as name, will not be disclosed in papers and presentations and will be announced in a way that participants cannot be identified. Also, if you request us, we will report you the analysis results of the questionnaire and the outline of the research results. Please feel free to send/ask us your requests/questions.
8. การเผยแพร่ผลงาน ความเป็นไปได้ของข้อเสนอแนะ
(รวมถึงทุกกรณีโดยไม่คำนึงถึงการระบุตัวบุคคล/ไม่สามารถระบุตัวบุคคลได้ในขณะที่นำเสนองานวิจัย)
ผลการวิจัยนี้จะประกาศเป็นผลสัมฤทธิ์ทางการเรียน
แต่ข้อมูลส่วนบุคคลเช่นชื่อจะไม่ถูกเปิดเผยในเอกสารและการนำเสนอและจะประกาศในลักษณะที่ไม่สามารถระบุผู้เข้าร่วมได้ นอกจากนี้ หากคุณร้องขอเรา เราจะรายงานผลการวิเคราะห์ของแบบสอบถามและโครงร่างของผลการวิจัยให้คุณทราบ โปรดส่ง / ถามถึงคำขอ / คำถามของคุณ

9. Confidentiality and handling of personal information (range of collaborators handling personal information, etc.)
Your display name will be used for matching questionnaire data with your game logs. We would not trace the identity of you. After this matching, Display names will be removed, and data to be used in research will be kept in the form that all individuals are anonymous. These data are used only for the period necessary for research, and under the responsibility of the research director.
9. การรักษาความลับและการจัดการข้อมูลส่วนบุคคล (กลุ่มผู้ทำงานร่วมกันในการจัดการข้อมูลส่วนบุคคล ฯลฯ)
ชื่อที่แสดงของคุณจะถูกใช้สำหรับจับคู่ข้อมูลแบบสอบถามกับบันทึกเกมของคุณ เราจะไม่ติดตามตัวตนของคุณ หลังจากการจับคู่นี้ ชื่อที่แสดงจะถูกลบออก และข้อมูลที่จะใช้ในการวิจัยจะถูกเก็บไว้ในรูปแบบที่บุคคลทั้งหมดไม่ระบุชื่อ
ข้อมูลเหล่านี้ถูกใช้ในช่วงเวลาที่จำเป็นสำหรับการวิจัยเท่านั้น และอยู่ภายใต้ความรับผิดชอบของผู้อำนวยการฝ่ายวิจัย

10. Handling of research data (Period during which disclosure/disposal (withdrawal of consent) can be requested, storage method/period, disposal time, etc.)
We will ask for your cooperation to provide your display name. Your display name will be required for matching your questionnaire data with your game logs for data analysis. Your display will be used to keep records on who has already played this game (in future experiments, we may look for students who have never played this game).
10. การจัดการข้อมูลการวิจัย (ระยะเวลาที่สามารถขอเปิดเผย/กำจัด (การเพิกถอนความยินยอม) วิธีการจัดเก็บ/ระยะเวลา ระยะเวลาในการกำจัด ฯลฯ)
เราจะขอความร่วมมือจากคุณในการระบุชื่อที่แสดงของคุณ
คุณจะต้องใช้ชื่อที่แสดงในการจับคู่ข้อมูลแบบสอบถามกับบันทึกเกมเพื่อการวิเคราะห์ข้อมูล
การแสดงผลของคุณจะถูกนำมาใช้เพื่อบันทึกว่าใครเคยเล่นเกมนี้มาก่อน (ในการทดลองในอนาคต เราอาจมองหานักเรียนที่ไม่เคยเล่นเกมนี้)

11. Existence and name of funding sources for research
This research was supported in part by Grant-in-Aid for Scientific Research (C), Number 19K12291, Japan Society for the Promotion of Science, Japan
11. การดำรงอยู่และชื่อแหล่งทุนวิจัย
งานวิจัยนี้ได้รับการสนับสนุนส่วนหนึ่งโดย Grant-in-Aid for Scientific Research (C), Number 19K12291, Japan Society for the Promotion of Science, Japan

12. Researchers and contact information
This research will be conducted by the following members.
Supervisor: Prof. Ruck Thawonmas, email: ruck@is.ritsumei.ac.jp
Researcher: Pujana Paliyawan, email: pujana.p@gmail.com
Please contact Pujana Paliyawan if you have any questions, requests, or concerns.
12. นักวิจัยและข้อมูลการติดต่อ
การวิจัยนี้จะดำเนินการโดยสมาชิกดังต่อไปนี้
หัวหน้างาน: Prof. Ruck Thawonmas, email: ruck@is.ritsumei.ac.jp
ผู้วิจัย : Pujana Paliyawan, email: pujana.p@gmail.com
โปรดติดต่อ Pujana Paliyawan หากคุณมีคำถาม คำขอ หรือข้อกังวลใดๆ

Agreement to participate in research
ข้อตกลงในการเข้าร่วมการวิจัย

* 1. I have received an explanation regarding “Audience Participation FIghtingICE,” understood the purpose, method, etc. of the research, understood the above points, and/but
ฉันได้รับคำอธิบายเกี่ยวกับ “Audience Participation FIightingICE” เข้าใจวัตถุประสงค์ วิธีการ ฯลฯ ของการวิจัย เข้าใจประเด็นข้างต้น และ/แต่

- ◯ Agree
  ตกลง
- ◯ Disagree
  ไม่ตกลง

to participate in the research.
เข้าร่วมการวิจัย

* 2. Participant (Full name)
ผู้เข้าร่วม (ชื่อเต็ม)

[ ]

* 3. Email

อีเมล
